# “I don’t want to die”: a qualitative study of coping strategies to prevent fentanyl-related overdose deaths among people who inject drugs and its implications for harm reduction policies

**DOI:** 10.1186/s12954-023-00805-x

**Published:** 2023-06-14

**Authors:** R. Abadie

**Affiliations:** Department of Anthropology, University of Nebraska-Lincoln, 839 Oldfather Hall, Lincoln, NE 68588 USA

**Keywords:** Fentanyl, Overdose, People who inject drugs, Harm reduction, Hurricane Maria, Puerto Rico

## Abstract

**Background:**

Fentanyl and fentanyl-related analogues are the main drivers of overdose death in the USA, particularly among people who inject drugs (PWID). Despite the fact that non-Hispanic whites exhibit higher population rates of synthetic opioid mortality, overdose deaths have increased among African American and Latinos in urban areas. Yet little attention has been paid to the introduction of fentanyl among rural PWID in Puerto Rico.

**Methods:**

We conducted *N* = 38 in-depth interviews with PWID in rural Puerto Rico to document participants’ experiences of injection drug use after the arrival of fentanyl and the strategies they implemented to manage overdose death risks.

**Results:**

Participants suggest that the arrival of fentanyl in large scale happened after Hurricane Maria in 2017; this coincided with a dramatic increase in overdose episodes and deaths. Fear of overdose deaths motivated some participants to substitute intravenous drug use for other forms of substance use or to seek MOUD. PWID that continued injection use resorted to conducting “hit tests,” avoiding injecting alone, using naloxone, and employing fentanyl testing strips.

**Conclusions:**

While overdose deaths would have been higher without participants’ willingness to adopt harm-reduction strategies, this paper illustrates the limits of these policies to address the current epidemic of fentanyl-related overdose deaths among this population. More studies are needed to understand how health disparities shape overdose risks for minority populations. However, major policy changes, in particular the revision of the harmful role of the War on Drugs and the termination of failed neoliberal economic policies that contribute to deaths of despair, should be addressed if we are to make a dent in this epidemic.

## Introduction

After a sharp increase starting in 2013 and a brief respite in 2018, opioid overdose deaths peaked again in 2020 [1–3] in the US Preliminary data show that in the past 12 months ending in October 2022 101,751 drug overdose deaths have been registered in the USA. Covid-19 seems to have aggravated this trend [4]. Fentanyl and fentanyl-related analogues are the main drivers of the overdose deaths in the USA. With a heavy presence in drug markets in the North East and MidWest, recently this synthetic opioid, 50 times more powerful than Morphine, has more recently reached the South and West US markets [7]. In addition, fentanyl contained in heroin seems to increasingly affect polysubstance users of stimulants like cocaine and methamphetamine [5, 6].

The arrival of fentanyl has radically modified the risk environment for people who inject drugs (PWID), increasing overdose risk [8]. Injecting in public places [9] and drug combining [10] are associated with higher overdose risk. Other factors for overdose risk include postincarceration, MOUD interruption, and polysubstance use [11]. Social and structural factors such as unemployment, poverty, homelessness, economic crisis or deindustrialization have also been linked with an increased risk of overdose deaths [12–14]. The presence of fentanyl and analogues magnified these risks, rising new challenges for the implementation of harm reduction policies.

Despite the terrible toll fentanyl-related deaths, particularly among PWID, relatively few studies have documented the ways in which people who use drugs have responded to the ongoing crisis. A study among opioid users conducted by McLean et al. in 2017 among opioid users in Southwest Pennsylvania shows that study participants were concerned by the risk of fentanyl-related overdose deaths adopting a series of strategies involving, substituting heroin for prescription opioids, abandoning heroin use and entering MOUD [15]. Other studies showed a willingness to employ Fentanyl Testing Strips, to assess drug quality [16, 17] or conducting tests hits to assess drug purity and intensity [18].

Confronted with epidemic levels of overdose episodes and deaths, PWID were required to implement, innovative, informal, community-based, and peer-to-peer harm reduction measures to respond to the overdose epidemic. Yet, little is known about how individual, behavioral and structural factors shaped PWID’s ability to enact these changes, particularly among rural PWID, or those from minority backgrounds who are disproportionally affected by fentanyl-related overdose deaths [19]. Despite the fact that non-Hispanic whites exhibit higher population rates of synthetic opioid mortality, overdose deaths have increased among urban African American and Latinos in urban areas [20]. In particular, overdose deaths among Puerto Ricans living in the USA are proportionally higher than among white non-Hispanics [21].

While fentanyl was already present in Puerto Rico a few months before Hurricane Maria, in September 2017, overdose deaths exploded in its aftermath [22]. By chance, we had been on the Island since 2015, conducting a multi-year study of social networks and HIV/HCV risk among PWID in rural Puerto Rico and were able to document the changes brought by the introduction of fentanyl in the drug environment. This observational study, allowed us to compare pre–post-fentanyl-related injection behaviors, as well as participants’ efforts to cope with the arrival of fentanyl. Employing a qualitative methodology, this paper explores participants’ experiences of injection drug use after the arrival of fentanyl, offering a unique perspective on coping strategies to prevent overdose deaths. In so doing, the paper not only contributes to illustrate how health disparities shape overdose deaths among PWID, but also, shows the possibilities and limits of harm reduction policies in the time of fentanyl.

Due to a paucity of reliable epidemiological data, it is not possible to estimate recent overdose deaths among PWID in Puerto Rico. Some sources note a sharp increase since 2017 [23], however, hurricane Maria, disrupted an already limited surveillance in the Island [24]. Anecdotal evidence from syringe exchange programs (SEP) who have a presence on the ground as well as our own observations shows a dramatic rise in fentanyl-related overdose deaths.

In addition, PWID in Puerto Rico are also disproportionately exposed to other drug-related harms. Historically, Puerto Rico hosts one of the highest incidences of HIV and hepatitis C (HCV) infection in the USA, with a significant proportion of those infections coming from drug use. In recent surveillance data, HIV prevalence among PWID in Puerto Rico was 11.6% (compared to 6.4% in the continental USA) [25] and more than 80% of current PWID in Puerto Rico were found to be infected with HCV [26]. A comparative longitudinal study of (urban) Puerto Rican PWID living in Bayamon (PR) and Puerto Ricans PWID living in New York (NY) concluded that the former were almost four times as likely to contract HIV, and once infected they showed a mortality rate that was three times as high as their mainland counterparts [27]. Disparities in infection rates and survival between NY and PR injectors in that study were associated with lower levels of HIV prevention and care service utilization in PR in comparison to NY. A mere 9.6% (compared to 37% in NY) of HIV positive PWID in the PR sample accessed HIV treatment, and only 34% (compared to 47% in NY) reported using an SEP to obtain sterile injecting equipment [28–30].

From economic crisis and despair, to a lack of MAT [31] syringe exchange programs, or incarceration rates for what are often non-violent drug-related crimes [32] many risk factors for overdose deaths are magnified in Puerto Rico. The arrival of fentanyl finds a population that that has been significantly impacted by a string of natural and economic disasters (including financial collapse during the summer of 2017 where the island was forced to declare bankruptcy and was placed by the US Congress under the administration of a fiscal board charged with managing its economy, and the back-to-back devastation of hurricanes Irma and Maria in the fall of 2017 [33]. According to the last US Census, almost half of the population of the island (44%) lives in poverty, three times the rate in continental USA [34]. Among PWID, poverty levels are even higher with almost half having been homeless at some point during the past year [35].

## Materials and methods

### Data

Data were gathered as part of a large multi-phase study of social networks and HIV/HCV risk among active PWID residing in four rural towns in central Puerto Rico conducted between 2015 and 2019. A modified NHBS survey about injection risk behaviors was applied, and participants were also tested for HIV and HCV at different intervals. Respondent driven sampling was employed to recruit the study population. Methodological issues related to this sampling strategy have been reported elsewhere [36, 37]. In 2018, in the aftermath of hurricane Maria, we conducted a follow-up study to document changes in injection risk practices within this population and in particular, the coping strategies PWID enacted to manage risks during the arrival of fentanyl in the Island.

### Measures

Our sample was composed of (*N* = 38) PWID who had been previously enrolled in the parent study. We employed a purposeful sample, including participants from different sociodemographic backgrounds, and with diverse injection risk profiles, from high injection frequency participants to low frequency, or no longer injecting. Mindful of the gendered nature of PWID in Puerto Rico, particularly in rural areas where nine out of ten PWID are men, [38] we oversampled to include more women (*N* = 5) in our study.

A semi structured questionnaire inquired about participants’ experiences after the irruption of fentanyl, particularly, in relation to the changing drug supply, their risk perception, the effects on injection practices, and the strategies they enacted to minimize overdose risk. In addition, despite the difficult fieldwork conditions after Hurricane Maria that left parts of the island without electricity for months, a rapid ethnographic assessment [39] was employed to register the lived experience of the arrival of fentanyl among rural PWID. During three months the author and two research assistants followed participants to shooting galleries and other venues where they use drugs to document injection practices after the arrival of fentanyl. In addition to observe possible changes in risk management, we also had the opportunity to accompany participants to their MOUD appointments, providing another opportunity to discuss motivations to enter or remain in treatment and the role of fentanyl in their decision making process. Finally, in all our interactions we paid close attention to the ways participants talked about fentanyl. One particular question we had was if participants talked about Fentanyl in a positive or negative way, and which terms did they use.

### Analytic approach

Interviews were transcribed and translated using a Deep L, a translation software program. Transcripts were then revised for accuracy by a bilingual research assistant. Ethnographic field notes were audio-recorded and then analyzing using a qualitative software program, MAXQDA. Upon completion of the survey questionnaire (described below), all participants were given $40; those who participated in an ethnographic follow-up, lasting around one hour received an additional $40. All participants signed an informed consent form. The study received IRB approval through the IRB at the University of Nebraska-Lincoln. To protect confidentiality and anonymity, all names reported in the results are pseudonyms.

## Results

### The arrival of fentanyl

While participants agree that fentanyl was already present in the drug supply before Hurricane Maria, in September 2017, they point to the aftermath of this natural disaster as the moment when fentanyl arrived. According to Orvi, a participant in the early 30’s, fentanyl “exploded after Maria” and currently “is in everything.” PWID in rural Puerto Rico recognized the presence of fentanyl as soon as it was introduced. While for some participants, fentanyl is indistinguishable from heroin at simple view, others believe that they can recognize fentanyl due to its odor: “it smells different, I can’t explain” (Tonito), but most agree that the main difference from heroin can be seen during drug preparation. Before the arrival of Fentanyl, heroin had a white, creamy color and while “cooked” during preparation, it would turn to a brownish tone. Fentanyl, according to participants adopts a very different color: “It gets really dark, like coca cola but darker. It seems cigarette ashes when you see it in powder, but when you through water in, it gets darker” (Papo). For others, “It is like you were cooking Nestquick” (Chillin). The preparation is so dark that participants struggle to distinguish the blood entering the syringe: “It cooks black, you look at the syringe and you don’t know which is the blood and which is the drug. They are the same colour!|” (Tato). This change in color was immediately evident to participants: “Something dark. I have never seen anything like this before!|” (Miguelito). While most participants identify fentanyl due to its dark color during preparation, some participants have seen fentanyl acquire other colors during drug preparation: “I associate [fentanyl] to this dark color. But I have also cooked it pinkish and I have seen it also green.” (Kiri). Other participants associate fentanyl not to a particular color, but to a range of colors. For example, Chino compares preparing fentanyl to M&Ms. The following conversation illustrates this point:

Chino: Like M&Ms, look at it! [sitting in the study van, in a parking lot. He extracts his cooker, a tiny, metallic cap provided by a local syringe exchange provider]. He extends the cooker to the interviewer who holds it in his hand.

Interviewer: I can see different colors, am I right?

Chino: Yes, like yellow, blue and red. Do you see how it colors the cooker?

Interviewer: Yes, I do. Can I see the filter? Is it stain it too?

Chino: I don’t have it. Somebody asked me to clean it and I gave it to him. Look at the cooker, it’s red there. And look at the bottom, it’s yellow, red, blue. Look at that blue dot. Did you see it? And do you see the red dot here too? [pointing to a very small red dot]. If I go right now and I buy a bag and I cook it right here, talking to you, you’ll see it because the colors form right there, really quick, pam! It’s like the M&Ms when you pick them up in your hands and they leave their stains. Like that!

It is possible that the differences in color might be at least in part due to the fact that fentanyl has many different formulations that might react in particular ways during drug preparation. Some participants resented the transition to fentanyl, they were not only suspicious of the fentanyl appearance, but also, its effects. For this group, fentanyl was not: “droga,” the local jargon for heroin. And while PWID already knew that the available heroin was far from pure, cut with anything from benzodiazepines to any imitating substance like talcum, milk or baking powder, they failed to perceive any fentanyl effects, as this participant explains: “It [Fentanyl] doesn’t do anything to you. Before I got up at 5 am and I would buy eight [heroin] bags and three of “perico” [cocaine] and right there, I used to spend $100. Now, I don’t even spend $20 or $30 a day. If I still use it’s for the habit, just to fee the needle or something. I really don’t know. (Rafa). This group believed that not only they could not get a “high” but also, that its effects would wane off earlier, forcing them to hustle more often in order to afford the next dose. In their view, this drug was a “racket” (Vitin) a way of cheating PWID out of their money.

The dealers cheat, you know? Users are already suffering due to an addiction and they [dealers] they are cheating them, selling them something that addicts are going to buy because they are desperate. Dealers play with users’ lives selling them something that might kill them. I get pissed off, just knowing that there might be people without a conscience, that don’t care about the addicts’ life and that they put money before life. Bah, there is nothing that can be done, the street is like that! (Zuli)

It is possible that different perceptions about the quality or potency of fentanyl among PWID were based on frequency of use, with those using more frequently having an appreciation for the effects while those that used fentanyl more sparsely might harbor a more negative view of its effects.

Yet, the main and most concerning aspect of the arrival of fentanyl according to participants was not the change in drug appearance and quality. Fentanyl caused a dramatic increase in overdose events and deaths. Within a few months after the arrival of fentanyl, most PWID in our study knew somebody in their social network that had a fentanyl-related overdose episode or had died as a result of an overdose. Kiomara summarizes the effects of fentanyl-related overdoses:

“There is people that has died with that.” Another participant provides a more detailed account of the impact of fentanyl on drug-related overdose deaths:

It was after the hurricanes that it went up [overdose] with the Fentanyl. Nobody knew what it was, and folks were used to shot up more than one bag and when they used Fentanyl then…Thirteen or fourteen died only in our town and like twelve hospitalized, intubated due to the Fentanyl right now. There were folks that came right out of jail, bought [drugs], used and died there! (Manny).

### Shooting up and the management of overdose risk

While participants were initially worried about the arrival of fentanyl, those that continued injecting, soon became habituated to it. It might have helped to provide a sense of continuity in the drug supply that drug pricing did not change, and the same drug sizes were maintained.

“The first time we saw it [Fentanyl] was in the $10 bags and it made an impact. Wow! What a big bag and this drug, so brown! We were worried but they [drug dealers] always give it to try it out and they tell you: “Be careful, it is strong.” You get used to it and now if we don’t get fentanyl, we don’t feel anything. The downside is that with Fentanyl the high is more intense, but the effect goes away faster. You immediately want more. If we get a bag without fentanyl, filled with heroin or just a bit of fentanyl, it is a bit clear. As soon as we see it, we get mad because we want the brown one with Fentanyl: I’ll need more because this shit won’t do anything” (Chillin).

Mindful of the overdose risk, these participants sometimes resorted to check drug potency, injecting incrementally, instead of delivering all the load at once. One participant explains the procedure: “I wait, I load little by little, I never load the full load. I go from 10 [ml] in 10, waiting 10 to 15 s in between to know what I am feeling and If I feel that it is fine, then I load another bit and then a bit more. I never had an overdose.” (Kiomara).

In addition, participants might seek to avoid injecting alone, in order to be able to be assisted in the event of an overdose. This strategy is reinforced by the used of naloxone, an opioid antagonist medication that has been made available by the only syringe exchange provider in the area. Ethnographic data shows that participants knew of naloxone as soon as it became available preferring the injection modality to the nasal one, due to the familiarity with the intravenous route of administration. Numerous participants administered naloxone to others in the course of an overdose, “bringing them back,” its use had some limitations. For example, not all participants carried naloxone with them, preferring to leave it at home. And while at home, many inject alone, a last “shoot” to sustain them before they could acquire their dose again. Pablito illustrate some of these barriers:

“I have dealt with folks that overdose and I have saved them. Right now I don’t have Narcan with me, it’s at home but just the other day I used with somebody from around here. I think he wasn’t using every day, he gave me 50 [ml] and he got another 40 and overdosed. I gave him to Narcan shoots I had with me. He didn’t have any Narcan. That’s why I wonder: if one day it happens to me, who is going to save me?

Through the same syringe exchange programs, PWID were introduced to fentanyl test strips (FTS), to assist them determine whether fentanyl was present in their drug of choice. FTS are paper strips used to detect the presence of fentanyl not only in the drug of choice, but also, the cooker or cotton used in its preparation. Similarly to a pregnancy test, if fentanyl is present, one line appears on the strip while two parallel lines signal absence. Our ethnographic observations of participants while injecting drugs and of their interactions with the staff of the syringe exchange while receiving the FTS show that participants welcomed the idea of using FTS and many used them to test their drug supply. However, in order to test drugs, participants had to hustle and acquire them first. Finding that a “bag” contained fentanyl before injecting was not perceived as a barrier to use. Participants’ in this situation prioritized “la cura” or avoiding the painful effects of heroin withdrawal, choosing to use this dose, than to lose and have to go to the trouble of finding the money and the having to go to the “Punto,” drug selling spot to buy it again. Furthermore, participants’ belief that fentanyl is everywhere—confirmed by their own experience and reinforced by the use of the FTS-would mean that discarding a dose with fentanyl would not prevent an overdose risk because the next shoot might contain fentanyl as well. The main use of the FTS seems to be to provide a valuable opportunity to conduct harm reduction. Staff at the syringe exchange, not only distributed the strips, but also, discussed with their clients, the overdose risks associated with fentanyl and how to adopt safer injection practices, not injecting the whole load at once, but in increments, not using alone and carrying naloxone.

These strategies are not mutually exclusive, and some participants have enacted all in an effort to prevent an overdose episode. Nanito, in his 50s, preferred to inject with somebody else: “even if I have to share drugs with him,” and never shoots the full load but: “little by little.” Yet, he overdosed two times, and, on both occasions, he was administered naloxone. “They call me the dead alive!”. Only a few months later, the died as a result of another overdose when the person he was injecting with, got scared and ran away without assisting him, illustrating the limits of these strategies to cope with the impact of Fentanyl.

### Radical strategies: quitting, seeking treatment, shifting drug use patterns

“Basura,” Trash; “Porqueria,” Rubbish; “Demonio,” demon; “asesina,” killer; “muerte,” death; participants’ association with Fentanyl were overtly negative, even for those that still use it. This might suggest that the arrival of fentanyl in Puerto Rico was not prompted by demand but rather by supply. Concerns over drug quality, but primarily, the fear of experimenting or dying of an overdose, produced important changes in injection behaviors among PWID. Some became habituated to fentanyl and continued injecting but adopting some measures to minimize overdose risk. Others quit injection drug use and entered MOUD, while others adopted a participant driven “Harm Reduction” program, replacing opioid use with other drugs of choice from marihuana to potpourri, as is locally known, to crack or benzodiazepines or alcohol, anything that would prevent them from overdose death. Caman, a participant that had used for more than twenty years summarized the main motivation behind these changes: “I don’t want to die” (Caman).

The main strategies deployed by participants are quitting intravenous drug use, substitute injection drug use by other substances and entering treatment.

Chuchan illustrates how undesirable drug effects and the fear of suffering overdose deaths might prompt PWID to quit injection drug use: “[Fentanyl] is like when you use something to put yourself to sleep, but it makes immediate effect, you feel the hit in your heart. Since I was using by vein, I could feel it, pum! I got scared. I always used at home, I was lying in bed and thought: “shit, I am gonna lie here, mom is going to find me dead in my room.” And that day I decided to quit, and I have not used again.” (Chuchan).

This participant had been in a Methadone program in the past, and after this episode decided to re-enter treatment again. However, some PWID in the study suggested that MOUD might not be as effective treating fentanyl dependence. Chino entered a Methadone program in an attempt to manage drug use after the introduction of Fentanyl but continued using at the same high frequency and finally abandoned treatment after a few weeks. According to him neither Suboxone nor Methadone are effective in treating fentanyl because “Fentanyl is much stronger [than Suboxone or Methadone]” (Chino). Another participant agrees: “it is not the same, it doesn’t block it. If you are in Methadone and you use Fentanyl you will still feel the shoot. People on Methadone are hooked up, they still feel sick. It’s messed up.” (Pablito).

Finally, other participants attempt to avoid fentanyl by quitting intravenous drug use and shifting substance use to other substances that do not present the same risk of overdose death. Polysubstance users, PWID can then resort to some of substances they were co-using during their careers. Some prefer marihuana to help them with sleep, appetite and relaxation, while others resort to alcohol use, crack cocaine or benzodiazepines. Potpourri, a cheap synthetic cannabinoid widely available in Puerto Rico, has been adopted by PWID who believe that it can help mask the powerful Fentanyl withdrawal symptoms.

## Discussion

Results suggest that PWID in rural Puerto Rico were concerned by fentanyl-related overdose deaths that spiked after the arrival of synthetic opioids in the Island and adopted a number of strategies to cope with overdose risks. Active participants adopted hit tests, employed fentanyl testing strips, carried naloxone and attempted to inject in the presence of somebody else. These findings are consistent with other studies [16, 40–44].

Without participants willing to use naloxone or adopt other proven harm reduction practices like using fentanyl strips, not injecting alone, or conducting hit tests, the number of overdose deaths would probably be much higher [45, 46]. However, as the latest figures on fentanyl-related overdose deaths illustrate, these measures do not seem to be enough to curb this epidemic. For example, using fentanyl testing strips in most cases only confirmed to participants that most doses carried fentanyl. Having gone through the trouble (and expense) to acquire the drug, most users chose to inject after confirming the presence of fentanyl in their drug of choice. This finding has been corroborated by other studies showing lower utilization rates for fentanyl strips among minority populations [47]. While PWID in our study had access to naloxone, they did not carry it with them all the time, reducing the opportunity to use it in the event of an overdose. Not injecting alone is reinforced by the cultural practice of caballo, when PWID acquire and then use drugs together [48], but study participants might prefer to use alone, particularly either in their “last hit” of the day or the first hit in the morning. For those participants that attempted substituting heroin, or fentanyl for other substances, while marihuana use has been found to have positive effects [49], other substances like crack, alcohol, benzodiazepines, or K2, are not without risks. For example, more attention should be paid to the potential harms of alcohol use in a population with a very high prevalence of HCV, while benzodiazepines use increase mortality risks among polysubstance users [50]. Finally, seeking MOUD in the time of fentanyl raises a number of challenges. MAT might not be as effective for fentanyl [51]. In addition, while interrupting MAT increases the risks of experiencing an overdose death, this outcome is magnified due to the presence of fentanyl [52, 53]. In this context, punitive policies that penalize non-compliant treatment users with program termination should be re-examined [54].

This study shows that effects of fentanyl among rural PWID have been particularly severe. While there are currently no epidemiological studies available, our data and other anecdotal evidence, in media and through syringe exchange programs, suggest that overdose deaths have disproportionately affected this community. Some studies conducted among Puerto Ricans living in the USA and in Puerto Rico suggest that health disparities might explain the high overdose deaths among this population [55–59]. We argue that fentanyl-related overdose deaths and other drug-related harms among PWID in Puerto Rico, cannot be understood simply as the product of the emergence of a powerful synthetic opioid flooding the market. As Merrill Singer has noticed in accounting for previous epidemics, fentanyl overdose deaths, might not be characterized as an epidemic, but a syndemic event, where a multitude of socio-cultural factors add to the effects of fentanyl to create a public health crisis [60]. In the case of Puerto Rico, an ongoing economic crisis, a lack of MOUD, high rates of incarceration for drug-related offenses contributed to overdose deaths. Other factors include the use of speedball—the preferred drug of choice for injectors in and from the Island [61]—because the contamination of cocaine with fentanyl [6, 62] constitutes another risk factor. Finally, health disparities can be compounded by stigma, preventing PWID from accessing or remaining in treatment [59, 63, 64]. Inter-generational injection drug use, in this population, like in other minority groups [65] constitutes another health disparity, adding to the individual and community costs associated with the arrival of fentanyl. The recent increase in fentanyl-related overdose deaths among African American and Latinos [66] point to the need to conduct further research on the individual and structural forces driving overdose deaths among minority populations in the USA.

While more resources should be devoted to harm reduction strategies in order to curb fentanyl-related overdose deaths in the USA, there is a need to address the harms caused by the War on Drugs [67], as well as, the effects of a failed neoliberal economic model that produces a marginalized and impoverished population, seeking in intravenous drug use, the release or gratification that the system denies them. A multi-pronged approach that combines well known and tested harm reduction interventions, more researches into the effects health disparities have on overdose deaths among minority PWID and the elimination of the war on drugs are needed to make a dent on our current epidemic of fentanyl-related overdose deaths.

## Limitations

The study has some limitations. As this study is based on a population of PWID in rural Puerto Rico, a US territory, findings about the responses to the arrival of fentanyl might not be transferable to other settings, for example, urban locations. Despite this limitation, this study is the first to document the effects of fentanyl and the coping strategies implemented by a population of rural PWID that had not been studied before, and its emphasis on the individual, behavioral, and structural factors that support or impede the adoption of harm reduction measures to manage the risks of overdose death represents a contribution to the field. Given the emergence of fentanyl-related overdose deaths in the rural USA, this study’s findings can be used to help shape and design harm reduction policies to limit the ravages of fentanyl-related deaths of PWID in rural areas.

## Conclusions

To manage the risks of fentanyl-related overdose deaths, PWID in rural Puerto Rico implemented peer-based strategies ranging from replacing intravenous drug use with other forms of substance use to seeking MOUD. Those PWID that continued injection use resorted to conducting “hit tests,” avoiding injecting alone, using naloxone, and employing fentanyl testing strips. While overdose deaths would have been higher without participants’ willingness to adopt such harm reduction strategies, findings illustrate the new challenges fentanyl poses to adopting a harm reduction approach, particularly for minority populations in rural settings.

## Data Availability

The interview transcripts generated during the study are not publicly available to preserve the confidentiality of the participants.

## Abbreviations

FTS: Fentanyl testing strips
HIV: Human Immunodeficiency Virus
HCV: Hepatitis C Virus
MOUD: Medication for Opioid Use Disorder
NHBS: National HIV Behavioral Survey
SEP: Syringe exchange programs
USA: United States of America

## References

[1] Ciccarone D (2019). The triple wave epidemic: supply and demand drivers of the US opioid overdose crisis. Int J Drug Policy.

[2] Hedegaard H, Miniño AM, Warner M. Drug overdose deaths in the United States, 1999–2018 key findings data from the national vital statistics system, Mortality. 1999. https://www.cdc.gov/nchs/products/index.htm.

[3] O’Donnell J, Gladden RM, Goldberger BA, Mattson CL, Kariisa M (2020). Notes from the field: opioid-involved overdose deaths with fentanyl or fentanyl analogs detected—28 states and the District of Columbia, July 2016–December 2018. MMWR Morb Mortal Wkly Rep.

[4] Wakeman SE, Green TC, Rich J (2020). An overdose surge will compound the COVID-19 pandemic if urgent action is not taken. Nat Med.

[5] Ciccarone D (2021). The rise of illicit fentanyls, stimulants and the fourth wave of the opioid overdose crisis. Curr Opin Psychiatry.

[6] Nolan ML, Shamasunder S, Colon-Berezin C, Kunins HV, Paone D (2019). Increased presence of fentanyl in cocaine-involved fatal overdoses: implications for prevention. J Urban Health.

[7] Zoorob M (2019). Fentanyl shock: the changing geography of overdose in the United States. Int J Drug Policy.

[8] Ciccarone D (2017). Fentanyl in the US heroin supply: a rapidly changing risk environment. Int J Drug Policy.

[9] Hunter K, Park JN, Allen ST, Chaulk P, Frost T, Weir BW, Sherman SG (2018). Safe and unsafe spaces: non-fatal overdose, arrest, and receptive syringe sharing among people who inject drugs in public and semi-public spaces in Baltimore City. Int J Drug Policy.

[10] Kline A, Mattern D, Cooperman N, Williams JM, Dooley-Budsock P, Foglia R, Borys S (2021). Opioid overdose in the age of fentanyl: risk factor differences among subpopulations of overdose survivors. Int J Drug Policy.

[11] Park JN, Weir BW, Allen ST, Chaulk P, Sherman SG (2018). Fentanyl-contaminated drugs and non-fatal overdose among people who inject drugs in Baltimore. MD Harm Reduct J.

[12] Case A, Deaton A (2017). Mortality and morbidity in the 21st century. Brook Pap Econ Act.

[13] Hollingsworth A, Ruhm CJ, Simon K (2017). Macroeconomic conditions and opioid abuse. J Health Econ.

[14] Pear VA, Ponicki WR, Gaidus A, Keyes KM, Martins SS, Fink DS, Cerdá M (2019). Urban-rural variation in the socioeconomic determinants of opioid overdose. Drug Alcohol Depend.

[15] McLean K, Monnat SM, Rigg K, Sterner GE, Verdery A (2019). “You never know what you’re getting”: opioid users’ perceptions of fentanyl in Southwest Pennsylvania. Subst Use Misuse.

[16] McGowan CR, Harris M, Platt L, Hope V, Rhodes T (2018). Fentanyl self-testing outside supervised injection settings to prevent opioid overdose: do we know enough to promote it?. Int J Drug Policy.

[17] Reed MK, Roth AM, Tabb LP, Groves AK, Lankenau SE (2021). "I probably got a minute": perceptions of fentanyl test strip use among people who use stimulants. Int J Drug Policy.

[18] Carroll JJ, Marshall BDL, Rich JD, Green TC (2017). Exposure to fentanyl-contaminated heroin and overdose risk among illicit opioid users in Rhode Island: a mixed methods study. Int J Drug Policy.

[19] Lippold KM, Jones CM, Olsen EO, Giroir BP (2019). Racial/ethnic and age group differences in opioid and synthetic opioid-involved overdose deaths among adults aged ≥18 years in metropolitan areas—United States, 2015–2017. MMWR Morb Mortal Wkly Rep.

[20] Wilson N, Kariisa M, Seth P, Smith H, Davis NL (2020). Drug and opioid-involved overdose deaths—United States, 2017–2018. MMWR Morb Mortal Wkly Rep.

[21] Cano M, Gelpí-Acosta C (2021). Drug overdose mortality among stateside Puerto Ricans: evidence of a health disparity. Int J Drug Policy.

[22] Rodríguez P. Cobra ocho vidas el fentanilo. El Vocero [Internet]. 15 July 2017. https://www.elvocero.com/gobierno/cobra-ocho-vidas-el-fentanilo/article_fffe8a4a-6907-11e7-a18b-abe061ee9959.html.

[23] Bauzá N. Peligrosa droga fentanilo ya está aquí. Primera Hora [Internet]. 23 March 2017. http://www.primerahora.com/noticias/policia-tribunales/nota/peligrosadrogafentaniloyaestaaqui-1213365/.

[24] Coto, D. Puerto Rico: La crisis de los opioides se siente con fuerza. Associated Press Spanish [Internet]. 7 January 2019. https://www.sun-sentinel.com/elsentinel/fl-es-crisis-opioides-con-fuerza-puerto-rico-20190107-story.html.

[25] CDC—HIV/AIDS—statistics and surveillance—reports—HIV surveillance report 2010. (n.d.).

[26] Abadie R, Dombrowski K (2020). “Caballo”: risk environments, drug sharing and the emergence of a hepatitis C virus epidemic among people who inject drugs in Puerto Rico. Harm Reduct J.

[27] Gelpí-Acosta C, Hagan H, Jenness SM, Wendel T, Neaigus A (2011). Sexual and injection-related risks in Puerto Rican-born injection drug users living in New York City: a mixed-methods analysis. Harm Reduct J.

[28] Andía JF, Deren S, Robles RR, Kang SY, Colón HM (2008). Peer norms and sharing of injection paraphernalia among Puerto Rican injection drug users in New York and Puerto Rico. AIDS Educ Prev.

[29] Robles RR, Colón HM, Matos TD, Finlinson HA, Muñoz A, Marrero CA, Reyes JC (1998). Syringe and needle exchange as HIV/AIDS prevention for injection drug users in Puerto Rico. Health Policy.

[30] Robles RR, Colón HM, Sahai H, Matos T, Marrero CA, Reyes JC (1992). Behavioral risk factors and human immunodeficiency virus (HIV) prevalence among intravenous drug users in Puerto Rico. Am J Epidemiol.

[31] Abadie R, Gelpi-Acosta C, Davila C, Rivera A, Welch-Lazoritz M, Dombrowski K (2018). “It Ruined My Life”: the effects of the War on Drugs on people who inject drugs (PWID) in rural Puerto Rico. Int J Drug Policy.

[32] Abadie R, Welch-Lazoritz M, Khan B, Dombrowski K (2017). Social determinants of HIV/HCV coinfection: a case study from people who inject drugs in rural Puerto Rico. Addict Behav Rep.

[33] Villanueva J, Cobián M, Rodríguez F (2018). San Juan, the fragile city: finance capital, class, and the making of Puerto Rico’s economic crisis. Antipode.

[34] U.S Census. Poverty: 2019 and 2021 [cited 11 January 2023]. https://www.census.gov/content/dam/Census/library/publications/2022/acs/acsbr22-014.pdf.

[35] Duncan I, Curtis R, Reyes JC, Abadie R, Khan B, Dombrowski K (2017). Hepatitis C serosorting among people who inject drugs in rural Puerto Rico. Prevent Med Rep.

[36] Habecker P, Abadie R, Welch-Lazoritz M, Reyes JC, Khan B, Dombrowski K (2017). Injection partners, HCV, and HIV status among rural persons who inject drugs in Puerto Rico. Subst Use Misuse.

[37] Hautala D, Abadie R, Khan B, Dombrowski K (2017). Rural and urban comparisons of polysubstance use profiles and associated injection behaviors among people who inject drugs in Puerto Rico. Drug Alcohol Depend.

[38] López LM, De Saxe Zerden L, Bourgois P, Hansen H, Abadie R, Dombrowski K, Curtis R (2015). HIV/AIDS in Puerto Rican people who inject drugs: policy considerations. Am J Public Health.

[39] Fessel J, Mars S, Bourgois P, Ciccarone D (2020). Inside ethnography: researchers reflect on the challenges of reaching hidden populations.

[40] Goldman JE, Krieger MS, Buxton JA, Lysyshyn M, Sherman SG, Green TC, Marshall BDL (2019). Suspected involvement of fentanyl in prior overdoses and engagement in harm reduction practices among young adults who use drugs. Subst Abuse.

[41] Krieger MS, Goedel WC, Buxton JA, Lysyshyn M, Bernstein E, Sherman SG, Marshall BDL (2018). Use of rapid fentanyl test strips among young adults who use drugs. Int J Drug Policy.

[42] Laing MK, Tupper KW, Fairbairn N (2018). Drug checking as a potential strategic overdose response in the fentanyl era. Int J Drug Policy.

[43] Latkin CA, Dayton L, Davey-Rothwell MA, Tobin KE (2019). Fentanyl and drug overdose: perceptions of fentanyl risk, overdose risk behaviors, and opportunities for intervention among people who use opioids in Baltimore, USA. Subst Use Misuse.

[44] Peiper NC, Clarke SD, Vincent LB, Ciccarone D, Kral AH, Zibbell JE (2019). Fentanyl test strips as an opioid overdose prevention strategy: findings from a syringe services program in the Southeastern United States. Int J Drug Policy.

[45] Rouhani S, Park JN, Morales KB, Green TC, Sherman SG (2019). Harm reduction measures employed by people using opioids with suspected fentanyl exposure in Boston, Baltimore, and Providence. Harm Reduct J.

[46] Reed MK, Guth A, Salcedo VJ, Hom JK, Rising KL (2022). "You can't go wrong being safe": motivations, patterns, and context surrounding use of fentanyl test strips for heroin and other drugs. Int J Drug Policy.

[47] Tilhou AS, Birstler J, Baltes A, Salisbury-Afshar E, Malicki J, Chen G, Brown R (2022). Characteristics and context of fentanyl test strip use among syringe service clients in southern Wisconsin. Harm Reduct J.

[48] Abadie R, Cano M, Habecker P, Gelpí-Acosta C (2022). Substance use, injection risk behaviors, and fentanyl-related overdose risk among a sample of PWID post-Hurricane Maria. Harm Reduct J.

[49] Socías ME, Choi JC, Lake S, Wood E, Valleriani J, Hayashi K, Milloy MJ (2021). Cannabis use is associated with reduced risk of exposure to fentanyl among people on opioid agonist therapy during a community-wide overdose crisis. Drug Alcohol Depend.

[50] Walton GRT, Hayashi K, Bach P, Dong H, Kerr T, Ahamad K, Wood E (2016). The impact of benzodiazepine use on mortality among polysubstance users in Vancouver, Canada. Public Health Rep.

[51] Silverstein SM, Daniulaityte R, Martins SS, Miller SC, Carlson RG (2019). “Everything is not right anymore”: buprenorphine experiences in an era of illicit fentanyl. Int J Drug Policy.

[52] Stone AC, Carroll JJ, Rich JD, Green TC (2018). Methadone maintenance treatment among patients exposed to illicit fentanyl in Rhode Island: safety, dose, retention, and relapse at 6 months. Drug Alcohol Depend.

[53] Stone AC, Carroll JJ, Rich JD, Green TC (2020). One year of methadone maintenance treatment in a fentanyl endemic area: safety, repeated exposure, retention, and remission. J Subst Abuse Treat.

[54] Harris J, McElrath K (2012). Methadone as social control: Institutionalized stigma and the prospect of recovery. Qual Health Res.

[55] Thrash C, Welch-Lazoritz M, Gauthier G, Khan B, Abadie R, Dombrowski K, RolonColon Y (2017). Rural and urban injection drug use in Puerto Rico: Network implications for human immunodeficiency virus and hepatitis C virus infection. J Ethn Subst Abuse.

[56] Cano M (2020). Drug overdose deaths among US hispanics: trends (2000–2017) and recent patterns. Subst Use Misuse.

[57] Cano M, Gelpí-Acosta C (2021). Risk of drug overdose mortality for Island-Born and US-Born Puerto Ricans, 2013–2019. J Racial Ethnic Health Disparities.

[58] Abadie R, Welch-Lazoritz M, Gelpi-Acosta C, Reyes JC, Dombrowski K (2016). Understanding differences in HIV/HCV prevalence according to differentiated risk behaviors in a sample of PWID in rural Puerto Rico. Harm Reduct J.

[59] Madden EF (2019). Intervention stigma: How medication-assisted treatment marginalizes patients and providers. Soc Sci Med.

[60] Singer M, Bulled N, Ostrach B, Mendenhall E (2017). Syndemics and the biosocial conception of health. The Lancet.

[61] Gelpí-Acosta C, Guarino H, Benoit E, Deren S, Pouget ER, Rodríguez A (2019). Injection risk norms and practices among migrant Puerto Rican people who inject drugs in New York City: the limits of acculturation theory. Int J Drug Policy.

[62] LaRue L, Twillman RK, Dawson E, Whitley P, Frasco MA, Huskey A, Guevara MG (2019). Rate of fentanyl positivity among urine drug test results positive for cocaine or methamphetamine. JAMA.

[63] Olsen Y, Sharfstein JM (2014). Confronting the stigma of opioid use disorder—and its treatment. JAMA.

[64] Varas-Díaz N, Santiago-Negrón S, Neilands TB, Cintrón-Bou F, Malavé-Rivera S (2010). Stigmatization of illicit drug use among puerto rican health professionals in training. P R Health Sci J.

[65] Myhra LL, Wieling E (2014). Intergenerational patterns of substance abuse among urban American Indian families. J Ethn Subst Abuse.

[66] Larrochelle MR, Slavova S, Root ED, Feaster DJ, Ward PJ, Selk SC, Knott C, Villani J, Samet JH (2021). Disparities in opioid overdose deaths trends by race/ethnicity, 2018–2019, from the HEALing Communities Study. Am J Pub Health.

[67] Socías ME, Wood E (2017). Epidemic of deaths from fentanyl overdose. BMJ.

